# Whole-genome sequence of *Staphylococcus epidermidis* strain AH3, isolated from Nile tilapia (*Oreochromis niloticus*)

**DOI:** 10.1128/MRA.00682-23

**Published:** 2023-10-06

**Authors:** Ahmed H. Al-Harbi

**Affiliations:** 1 Sustainability and Environment Sector, King Abdulaziz City for Science and Technology, Riyadh, Saudi Arabia; Loyola University Chicago, Chicago, Illinois, USA

**Keywords:** genome sequence, *Staphylococcus epidermidis*, tilapia, illumina, pacific Biosciences

## Abstract

The whole-genome sequence of *Staphylococcus epidermidis* strain AH3 isolated from moribund farmed Nile tilapia (*Oreochromis niloticus*) was performed using a combination of the Illumina and Pacific Biosciences (PacBio) sequencing platforms. The genome sequence is composed of a single chromosome of 2,464,380 bp with a GC content of 32.2% and 2,220 predicted protein-coding genes.

## ANNOUNCEMENT

*Staphylococcus epidermidis* is a Gram-positive bacterium that is a commensal microorganism of the skin and mucous membranes of humans and other mammals (1). However, this bacterial species has also been reported previously as a fish pathogen in some freshwater and marine fish (2 – 5).

*S. epidermidis* strain AH3 was originally isolated in 2003 from the kidney of moribundfarmed Nile tilapia (*Oreochromis niloticus*) in Riyadh, Saudi Arabia, as described previously (6). For DNA isolation, *S. epidermidis* strain AH3 was streaked onto tryptone soy agar (Oxoid) from frozen stocks at −80°C and incubated overnight at 30°C. A single colony was inoculated into 10 mL of tryptone soy broth (Oxoid) and grown aerobically overnight at 30°C. Bacterial cells were collected by centrifugation, and the genomic DNA was extracted using the DNeasy blood and tissue kit (Qiagen) following the manufacturer’s instructions. The DNA quantity and quality were assessed using a NanoDrop spectrophotometer (Thermo Fisher Scientific, USA) and 1% agarose gel electrophoresis, respectively.

Purified genomic DNA was sequenced using both Illumina and Pacific Biosciences (PacBio) platforms. For Illumina short-read sequencing, genomic DNA was fragmented to an average size of ~470 bp using g-TUBEs device (Covaris, Woburn, MA, USA). Libraries were generated with NEBNext Ultra II DNA Library Preparation Kit (New England Biolabs, Ipswich, MA, USA), visualized on a 2,100 Bioanalyzer (Agilent Technologies, Inc., Santa Clara, CA, USA), and quantified by quantitative PCR with a KAPA Library Quantification Kit (Thermo Fisher Scientific). Paired-end (2 × 150 bp) reads were performed on the NovaSeq 6,000 platform (Illumina Inc., USA). Trimmomatic v0.30 was used to trim adapters from the Illumina reads with a sliding window quality cutoff score of Q15 (7). For PacBio long-read sequencing, genomic DNA was sheared using g-TUBEs device (Covaris), size selected to ≥10 kb using the BluePippin system, end repaired and ligated with universal hairpin adapters using the SMRTbell Template Preparation Kit v1.0 (PacBio, Menlo Park, CA, USA) following the manufacturer’s instructions, and sequenced on the RSII platform (PacBio) using P6-C4 chemistry and 240-min movies. The genome sequences were assembled *de novo* using Flye v2.8 (8) and Mecat2 vJAN-2020 software run with default parameters. The assembly was further error-corrected and polished using Pilon v1.22 (9). The quality and completeness of the assembled genome were assessed by Benchmarking Universal Single-Copy Orthologs v4.1.4 (10) with the bacteria_odb10 data set. This resulted in 100% complete genes, with 100% of the genome being single-copy genes, 0.0% duplicated genes, 0.0% fragmented genes, and 0.0% missing genes.

The genomes were annotated by NCBI using the Prokaryotic Genome Annotation Pipeline v4.13 (11, 12). Default parameters were used for all software unless otherwise specified.

The *S. epidermidis* strain AH3 consists of six contigs with a total length of 2,638,649 bp and 2,341 total predicted protein-coding genes, with an N50 value of 13,316 bp and a GC content of 32.3%. The genome size and number of predicted protein-coding genes of the strain AH3 range from 6,531 bp to 2,464,380 bp and 4 to 2,220, respectively. The chromosome was determined to be 2,464,380 bp long, with a GC content of 32.2%. The assembly statistics and genomic features are shown in Table 1.

**TABLE 1:** Genomic characteristics of *Staphylococcus epidermidis* strain AH3

Characteristic	Value
Sequencing and assembly features	
No. Illumina reads	7,874,640
No. PacBio reads	447,037
Total no. of Illumina bases	1,162,156,500
Total no. of PacBio bases	5,447,067,835
Avg genome coverage (×)	1,785.94
	
Genome features	
Genome size (bp)	26,38,649
No. of contigs	6
GC content (%)	32.3
Total no. of genes	2,531
No. of coding sequences	2,417
No. of proteins	2,341
No. of pseudogenes	76
No. of rRNAs	22
No. of tRNAs	88
No. of noncoding RNAs	4

## Data Availability

The whole-genome sequence of *Staphylococcus epidermidis* strain AH3 has been deposited in DDBJ/ENA/GenBank under accession number JAQPSG000000000, BioProject accession number PRJNA802702, and BioSample accession number SAMN25580685. The version described in this paper is version JAQPSG010000000. The raw sequence reads were deposited in the Sequence Read Archive (SRA) under accession numbers SRR17857547 (Illumina) and SRR17858480 (PacBio).
